# Sleep quality of endometrial cancer survivors and the effect of treatments

**DOI:** 10.4274/tjod.59265

**Published:** 2017-12-30

**Authors:** Tolgay Tuyan İlhan, Mustafa Gazi Uçar, Ayhan Gül, Türkan Saymaz İlhan, Güler Yavaş, Çetin Çelik

**Affiliations:** 1 Mersin Maternity and Children’s Diseases Hospital, Clinic of Obstetrics and Gynecology, Mersin, Turkey; 2 Selçuk University Faculty of Medicine, Department of Obstetrics and Gynecology, Konya, Turkey; 3 Selçuk University Faculty of Medicine, Deparment of Radiation Oncology, Konya, Turkey

**Keywords:** Endometrial cancer, sleep disorders, sleep quality

## Abstract

**Objective:**

Sleep disorders affect 54.9% of gynaecologic cancer survivors. The effect of treatment methods on sleep quality is not clear. This study evaluated the sleep quality of survivors of endometrial cancer and compared the effects of different treatments on sleep quality.

**Materials and Methods:**

Patients were categorised as surgery (group 1), surgery + brachytherapy (BRT) (group 2), surgery + external beam radiation therapy (EBRT) (group 3), and surgery + EBRT + BRT + chemotherapy (group 4). Sleep quality was assessed using the Pittsburgh Sleep Quality Index (PSQI) questionnaire. The PSQI was completed by the participants before surgery, 1, 3, and 6 months after each treatment was completed. The PSQI scores were compared between the different measurement times and different study groups.

**Results:**

This study enrolled 114 patients with a mean age of 58.1±11 years. The number of participants in each group was 53 (46.5%), 14 (12.3%), 12 (10.5%), and 35 (30.7%), respectively. At baseline, 28 (24.6%) patients reported poor sleep quality. The mean PSQI score reached the maximum level at the second measurement and decreased slightly during follow-up and the change in the PSQI score was significant (p=0.001). Group 3 and group 4 had significantly higher scores from baseline (p<0.008). At time point 3, the differences between the groups were significant. At time point 4, the most prominent effect of treatment on sleep quality was observed in patients with combined chemo-radiotherapy when compared with the other study groups.

**Conclusion:**

Most survivors of endometrial cancer are affected by poor sleep quality during their treatment. To improve these patients’ quality of life, this disorder must be considered at each visit and tailored care plans should be developed to meet the women’s needs. Further studies are needed to evaluate the long-term results of sleep quality on patients with endometrial cancer.

## PRECIS:

We determined the effect of treatment on the sleep quality of endometrial cancer survivors by using special questionnaire.

## INTRODUCTION

Sleep disorders affect an estimated 35% to 40% of adults^(1)^. These disorders affect 54.9% of survivors of gynaecologic cancer and constitute a prevalent health issue^(2)^. Sleep disorders have been shown to adversely affect the quality of life (QoL) of patients with ovarian, breast, and lung cancer^(3,4,5,6)^. Sleep disorders may arise at any stage of cancer as both cancer itself and therapeutic modalities may lead to sleep disturbance. Several mechanisms have been hypothesized for sleep disturbance in patients with cancer. Some inflammatory signals, tumour necrosis factor and C-reactive protein, as well as the effects of depression and distress are postulated as mechanisms of sleep disorders^(7,8,9,10)^. It was previously shown that radiotherapy affects the QoL of women with endometrial cancer (EC) probably due to radiotherapy-induced urologic symptoms in patients with cervical cancer^(2,11)^. Moreover, surgery seems to affect sleep quality mainly through surgical stress, pain, and medications. The role of chemotherapy remains controversial so far with few studies reporting inconsistent results. However, the impact of specific types of cancer on sleep still needs to be addressed because several important cofactors for sleep disorders such as age and sex may vary in particular cancer types.

EC is the second most common gynaecologic malignancy worldwide,^(12)^ with an annual incidence of 300.000^(13)^. With improvements in diagnostic techniques and novel treatment modalities, most uterine cancers are diagnosed at an early stage, and patients have favourable prognoses^(14,15,16)^. So far, there are only robust data on the impact of EC on sleep quality. Therefore, the present study evaluated the sleep quality of EC survivors and compared the effects of different treatment modalities on sleep quality. In particular, we sought to determine if chemotherapy, radiotherapy or both as an adjuvant to surgery had any impact on sleep quality in patients with EC.

## MATERIALS AND METHODS

This study enrolled 114 patients with histologically proven EC who had completed treatment in the Selçuk University Faculty of Medicine Department of Gynaecological Oncology from 2012 to 2016. The study was approved by the Research Ethics Committee of Selçuk University (approval number: 2014/135). Written informed consent was obtained from all patients. All patients underwent a hysterectomy, bilateral salpingo-oophorectomy, and lymph node dissection. Debulking was performed as indicated for advanced-stage EC. All patients were staged according to the International Federation of Gynaecology and Obstetrics 2009 staging system for EC. For patients with stage Ia (<50% myometrial invasion) and grade 1 or 2 disease, adjuvant brachytherapy (BRT) was planned based on various risk factors, including patient age, lymphovascular space invasion, tumour size, and the presence of lower uterine (cervical glandular/stromal) infiltration. For women with grade 1 or 2 cancer and ≥50% myometrial invasion or grade 3 cancer and <50% myometrial invasion, vaginal BRT was performed. Patients with grade 3 cancer and ≥50% myometrial invasion or cervical stromal invasion were treated with external beam radiotherapy (EBRT). For women with high-risk early-stage disease or advanced disease, we administered EBRT ± BRT ± chemotherapy. Patients received concurrent paclitaxel (175 mg/m^2^) and carboplatin (AUC=5), every 3 weeks for 6 cycles. Patients who received only BRT postoperatively were treated in five fractions at a dose of 5.5 Gy/fraction. Patients with EC who were treated with EBRT received 45.0 to 50.4 gray (Gy) with three-dimensional conformal radiotherapy using 18 mega volt photon beams. When EBRT and BRT were combined, we used the same EBRT dose with three fractions of BRT at a dose of 7 Gy/fraction. The inclusion criteria were histologically proven EC and age of >18 years. The exclusion criteria were recurrent EC, preoperative sleep disorder, the use of antipsychotic or anxiolytic drugs, and loss to follow-up. Demographic data, physical properties, medical comorbidities, and complications were recorded.

### Measurement

Sleep quality was assessed using the Pittsburgh Sleep Quality Index (PSQI), which comprises 19 self-rated items and incorporates 7 different components (subjective sleep quality, sleep latency, habitual sleep efficiency, night-time disturbances, sleep duration, use of sleep medications, and daytime dysfunction); the total score is the sum of the component scores. A total score of >5 indicates poor sleep quality. This questionnaire is an effective instrument for assessing the sleep quality of patients with cancer^(17)^. The PSQI was first completed by the participants before surgery (time 1). The time of the second questionnaire differed for each treatment method. Results were obtained after the final pathologic examination, and the patients were assigned to different treatment groups: surgery (group 1), surgery + BRT (group 2), surgery + EBRT (group 3), and surgery + EBRT + BRT + chemotherapy (group 4). The second measurement was made 1 month after each treatment was completed (time 2), and the test was repeated 3 (time 3) and 6 (time 4) months after treatment was completed. The PSQI scores were compared at different measurement times in the different study groups.

### Statistical Analysis

Continuous variables were examined for normal distribution using the Kolmogorov-Smirnov test. Data are shown as the median (range) or number of cases and percentages where applicable. The mean values were compared between the groups using Student’s t-test, and the Mann-Whitney U test was used to compare median values. Nominal data were analysed using Pearson’s chi-square or Fisher’s exact test where applicable. Friedman’s test was used to determine the statistical significance of differences among repeated clinical measurements. When the Friedman test yielded a significant p-value, Wilcoxon’s 3 was used to identify which parameter was different. Bonferroni correction was applied to control for type 1 errors. Correlation analyses were performed to determine the correlations between sleep quality and variables. In all analyses, p<0.05 was taken to indicate statistical significance. The statistical analyses were performed with SPSS ver. 17.0 (SPSS, Chicago, IL).

## RESULTS

This study enrolled 114 patients with a mean age of 58.1±11 years. The final treatment was surgery for 53 (46.5%) patients, surgery + BRT for 14 (12.3%), surgery + EBRT for 12 (10.5%), and surgery + computed tomography + EBRT + BRT for 35 (30.7%). Ninety patients (78.9%) had type 1 EC and 24 (21.1%) had type 2 EC. The patients’ characteristics are shown in Table 1.

### Pittsburgh Sleep Quality Index scores

At baseline, 28 (24.6%) patients reported poor sleep quality (PSQI score of >5). There were no relationships between sleep quality and age, marital status, education status, economic status or menopausal status. A negative correlation was observed between body mass index and sleep quality (r=-0.189; p=0.4).

The mean PSQI score at time 1 was 4.59±2.80. The difference in the baseline PSQI score among the treatment groups was not significant (p=0.296). The score decreased slightly during follow-up. The change in the PSQI score was significant at each time point (p<0.05). Table 2 shows the mean PSQI score and rate of sleep disorders of the patients at the different measurement times.

At time 2, the PSQI score was 8.12±4.30, which was the maximum score reached. Poor sleep quality was reported by 79 (69.3%) patients. The differences between the study groups were significant (p<0.05). The difference between groups 1 and 2 was not significant. Group 3 had a higher PSQI score, but this was not significant. However, group 4 had a significantly higher score than the other groups (p<0.008) (Table 3). The rate of poor sleep quality was 60.4%, 64.3%, 66.7%, and 85.7% in groups 1 to 4, respectively. Patients in group 4 were more likely to have poor sleep quality than those undergoing other treatments [odds ratio (OR): 3.67; 95% confidence interval (CI): (1.28-10.49); p=0.011]. Subjective sleep quality, habitual sleep efficiency, and sleep duration were the most affected components of the PSQI in group 4.

At time 3, the mean PSQI score was 7.2±3.9. The differences between the treatment groups were significant (p=0.001). Group 4 had a significantly higher PSQI score than groups 1 and 2 (p<0.008). At time 3, the mean PSQI score in groups 1 and 2 was not significantly different from the baseline score, whereas groups 3 and 4 had higher scores (p<0.008) (Table 3). The rates of poor sleep quality were 22.6%, 35.6%, 66.7%, and 74.3% in groups 1 to 4, respectively. Patients in group 4 were more likely to have poor sleep quality than those receiving other treatments [OR: 6.24; 95% CI: (2.55-15.25); p≤0.001].

**Figure 1:**
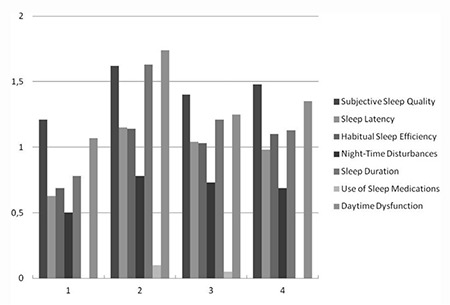
Component scores at each measurement time

At time 4, the mean PSQI score was 6.67±3.60. The differences between the treatment groups were significant (p=0.012). Group 4 had a significantly higher PSQI score than groups 1 and 2 (p<0.008), and group 4 had significantly poorer sleep quality (Table 3). Figure 1 shows the component scores and changes at each measurement time.

## DISCUSSION

To our knowledge, this is the first prospective study to evaluate sleep disorders and anxiety in Turkish women with EC. We followed up 114 patients for 6 months after the primary treatment was completed. The main findings of the present study are as follows: patients with EC have similar rates of sleep disturbance at diagnosis. Overall, sleep quality increases upon the completion of therapy, which then decreases gradually over time. Combined use of chemotherapy and radiotherapy has the greatest impact on sleep quality, but BRT had on adverse effect on sleep quality.

It was suggested in several studies that release of several cytokines such as tumour necrosis factor and C-reactive protein, as well as the effects of depression and distress were the mechanisms of sleep disorders. There is also a relationship between sleep disorders and cancer-related fatigue^(18)^, probably by desynchronisation of the circadian rhythm^(19)^. The relationship between noncancerous gynaecological conditions and poor sleep quality was observed in 33.7% of the participants with benign gynaecologic disease^(20)^. The importance of QoL on the prognosis of different cancer types was assessed in a global study and the authors reported that QoL predicted clinical outcomes well^(21)^. Before surgery, patients with ovarian cancer reported a high sleep disorder rate^(22)^. In ovarian cancer, abdominal discomfort might lead to poor sleep quality. Unlike previous studies, our data suggest that patients with EC had comparable sleep quality before treatment. In our study population, 24.6% of women had poor sleep quality before treatment, similar to that of the healthy population. We believe this difference may be related to the nature of EC which, unlike ovarian cancer, is generally localised at diagnosis and lacks abdominal symptoms.

Many studies have suggested an association between surgery and poor sleep quality^(23,24)^. Sleep fragmentation and reduced sleep time are the primary symptoms observed after surgery. Surgical stress, pain, and medications are suggested causes of poor sleep quality^(23,24)^. In our study cohort, the mean PSQI score increased significantly compared with the baseline scores and 79 (69.3%) patients had poor sleep quality postoperatively. Subjective sleep quality, sleep disturbances, and daytime dysfunction are the most affected components of the PSQI by surgery. Our data are in keeping with these observations because our cohort had the highest rate of sleep disturbance immediately after surgery (time 2).

Radiotherapy affects the QoL of women with EC, as shown in many studies^(2,11)^. There is an association between poor sleep quality and radiotherapy in patients with cervical cancer^(25)^. The potential mechanisms of the poor sleep quality after radiotherapy could be that the radiotherapy induces urologic symptoms^(2)^. Assessment of bladder symptoms in cervical and EC survivors showed that radiotherapy for EC was associated with nocturia, and BRT was associated with the severity of the nocturia^(26)^. In the present cohort, patients receiving EBRT alone as an adjuvant therapy had PSQI scores similar to patients receiving BRT or no adjuvant therapy. Daytime dysfunction, sleep latency, and subjective sleep quality were the components most affected after EBRT. Another study showed the impact of radiotherapy on the QoL of patients with cancer and concluded that the negative effect of radiotherapy was temporary and improved 1 month after treatment was completed^(27)^. In our study cohort, the PSQI scores were improved slightly at 3 months, but this was not significant. Six months after treatment, the improvement was significant. This result was similar to previous studies. The effect of radiotherapy on sleep quality continues for at least 3 months after radiotherapy is completed.

The effect of chemotherapy on sleep quality is controversial. In breast, prostate, and ovarian cancer, high rates of sleep disorders before treatment and improvement after treatment have been reported^(4,28)^. Poor sleep quality during chemotherapy for rectal cancer has also been reported^(29)^. Palesh et al.^(30)^ assessed the association between cancer and sleep quality and concluded that the rate of insomnia was three times higher in patients receiving chemotherapy. Patients receiving combined chemo-radiotherapy are more likely to develop sleep disorders. In our study, The PSQI scores were significantly higher in the chemo-radiotherapy group at all follow-up times. In the chemo-radiotherapy group, subjective sleep quality, habitual sleep efficiency, and sleep duration were the most affected components of the PSQI. We believe the adverse effect profile of the present chemotherapy regimen such as neurotoxicity, emesis, and nephrotoxicity might account for decreased sleep quality in patients who received chemotherapy.

### Study Limitation

Our study has some limitations that deserve mention. First, it was conducted at a single centre and the patient group was relatively small and heterogeneous. We did not evaluate symptoms that might affect sleep quality. Moreover, treatments could not be randomly assigned and sleep quality was assessed by only subjective sleep measures. However, this study was a prospective study and allowed for comparison different treatment methods.

## CONCLUSION

Most survivors of EC are affected by poor sleep quality during their treatment. The effects of surgery and BRT on sleep quality appear to be short-term effects. Combined treatment with radiotherapy and chemotherapy seemed to have more severe, long-term effects on sleep quality. This conclusion is based on changes in sleep quality scores at each timeline during treatment. However, we were unable to identify whether the higher rate of sleep disturbance was due to more advanced disease or the therapy itself. Further studies are needed to evaluate the long-term results of sleep quality on patients with EC.

## Figures and Tables

**Table 1:**
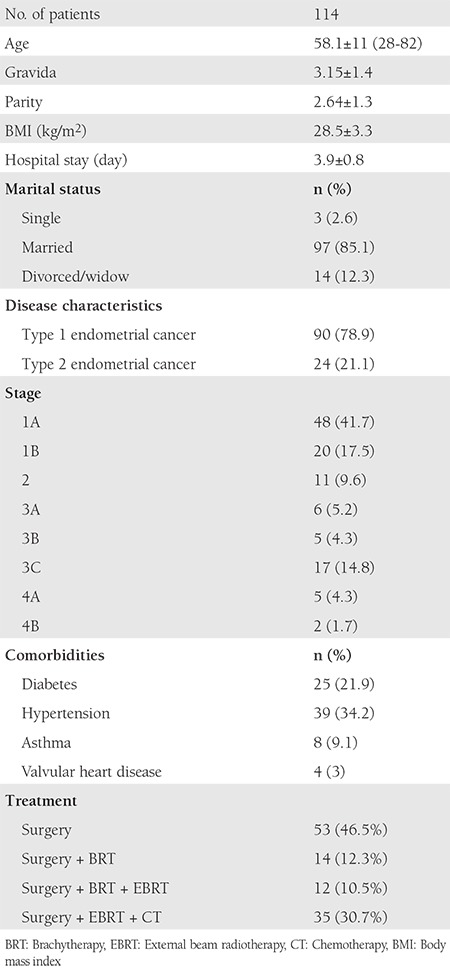
Patient’s characteristics

**Table 2:**
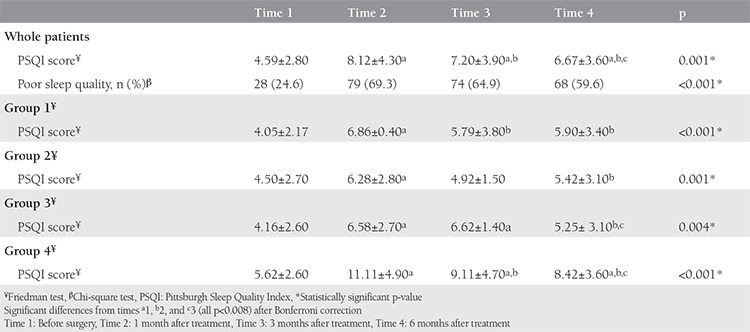
Comparison of Pittsburgh Sleep Quality Index scores at different measurement times

**Table 3:**
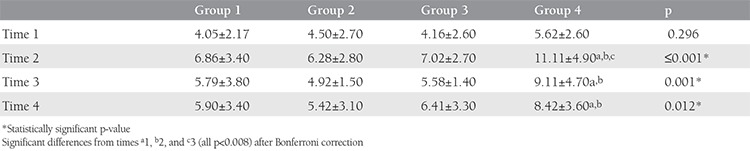
Comparison of Pittsburgh Sleep Quality Index scores of different treatment groups
